# 侵袭性系统性肥大细胞增多症伴RUNX1::RUNX1T1融合基因阳性急性髓系白血病1例

**DOI:** 10.3760/cma.j.cn121090-20251121-00544

**Published:** 2026-01

**Authors:** 渊博 刘, 香丽 陈, 磊 张, 玉龙 李, 成业 邬, 薇 程, 益民 刘, 妍伸 李, 尊民 朱

**Affiliations:** 河南省人民医院血液病研究所、郑州大学人民医院血液内科，郑州 450003 Institute of Hematology, Henan Provincial People's Hospital, Zhengzhou University People's Hospital, Zhengzhou 450003, China

患者，男，16岁，因“双下肢疼痛1个月余”为主要表现就诊于当地医院，血常规提示WBC明显升高、贫血、PLT明显减低。疑诊“急性髓系白血病（AML）”就诊于我院。查体：轻度贫血貌。全身皮肤黏膜无黄染，无皮疹、皮下出血，全身浅表淋巴结无肿大；胸骨无压痛，肝、脾肋缘下未触及。WBC 21.76×10^9^/L，HGB 88.0 g/L，PLT 8×10^9^/L，ALT 51.8 U/L。外周血涂片提示易见原始粒细胞及各阶段幼稚粒细胞。超声提示脾大。骨髓细胞形态学：增生极度活跃，原始粒细胞占30.4％，肥大细胞占1.6％，红系增生减低，巨核细胞少见。流式细胞术（FCM）表型分析：异常髓系幼稚细胞占有核细胞的13.46％，表达CD34、HLA-DR、CD33，部分表达CD38、CD117、CD13、CD123、CD56、MPO；另一群细胞占有核细胞3.32％，表达CD117^bri^、CD13、CD25，部分表达CD2、CD38、CD56，为异常表型肥大细胞。染色体核型分析：46，XY，t（8；21）（q22；q22）[8]/46，XY[22]。诊断为AML。行HAA方案（高三尖杉酯碱2 mg/d，第1～7天；阿糖胞苷150 mg/d，第1～7天；阿克拉霉素20 mg/d，第1～7天）化疗1个周期。

化疗后骨髓细胞形态学：增生极度活跃，肥大细胞占42.8％，原始粒细胞占6.4％，巨核细胞少见。FCM提示异常髓系幼稚细胞占有核细胞6.91％，表达CD34、HLA-DR、CD13^dim^、CD33^dim^、CD38、CD56，部分表达CD117；另一群细胞占有核细胞14.52％，表达CD117^bri^、CD13、CD33^bri^、CD25，部分表达CD7/2、CD38、CD56，为异常表型肥大细胞。骨髓组织病理学：骨髓活检可见大量不典型肥大细胞浸润，网状纤维染色（MF＝0）；骨髓组织免疫组化表现为CD117、类胰蛋白酶（Tryptase）、CD25、CD2、MPO阳性（图1）。复查染色体核型仍为异常核型；荧光原位杂交（FISH）：骨髓异常肥大细胞及原始粒细胞存在t（8；21）（q22；q22），即RUNX1::RUNX1T1融合基因阳性（图2）。融合基因筛查结果为RUNX1::RUNX1T1、WT1阳性；二代基因测序结果为KIT D816H［等位基因突变频率（VAF）33.10％］、TET2 G1172fs（VAF 10.00％）、EZH2 S21fs（VAF 42.90％）、SH2B3 R398C（VAF 48.90％）、SAMD9 N720fs（VAF 45.50％）、TTN T11933N（VAF 48.60％）、SAMD9 R106C（VAF 48.30％）。修正诊断为“侵袭性系统性肥大细胞增多症-AML”，进展型系统性肥大细胞增多症（AdvSM）的突变校正危险度评分（MARS）2分，中危组，继续行阿伐替尼（100 mg/d）+IA方案（去甲氧柔红霉素15 mg/d，第1～3天；阿糖胞苷150 mg/d，第1～7天）化疗2个周期、阿伐替尼（100 mg/d）+HAA方案化疗1个周期，治疗期间患者异常肥大细胞呈下降趋势，但细胞形态学、微小残留病、基因学始终未缓解。随访5个月后，患者复查骨髓细胞形态学可见异常肥大细胞占28.4％，提示疾病进展。

**图1:**
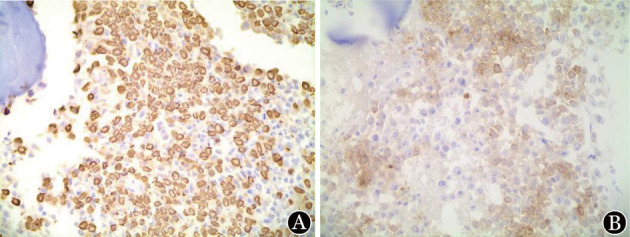
患者骨髓组织病理学及免疫组化结果（高倍镜视野） **A** Tryptase；**B** CD25

**图2:**
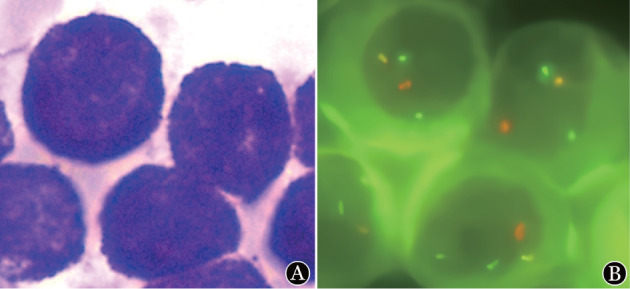
患者骨髓象（A）及对应区域荧光原位杂交检测结果（B）

讨论：SM是一种具有独特病理、临床特征和分子表达的髓系肿瘤类型，表现为侵袭性肥大细胞在真皮组织以外的一个或多个组织、器官或系统的克隆性增殖，并释放大量的血管活性介质，引起多器官功能障碍，常见的受累部位包括骨髓、皮肤、脾脏、淋巴结、肝脏和（或）单核-巨噬细胞系统。本例患者初诊时骨髓肥大细胞浸润不明显，抗白血病化疗后骨髓大量异常肥大细胞浸润，t（8;21）（q22;q22）探针行FISH检测发现原始粒细胞、肥大细胞均为阳性，可能SM和AML起源于相同的早期造血细胞，随后发展成表型不同的克隆，此结果与Pullarkat等研究结论相似。该患者进行骨髓组织免疫组化检测，表现出CD117、Tryptase、CD25、CD2阳性，证实该患者骨髓肥大细胞为异常表型。阿伐替尼是选择性KIT exon17抑制剂，该患者应用阿伐替尼治疗，表现出短暂的治疗反应后疾病迅速进展。

